# Fear, Risk, and the Responsible Choice: Risk Narratives and Lowering the Rate of Caesarean Sections in High-income Countries

**DOI:** 10.3934/publichealth.2017.6.615

**Published:** 2017-12-26

**Authors:** Helga Hallgrimsdottir, Leah Shumka, Catherine Althaus, Cecilia Benoit

**Affiliations:** 1 School of Public Administration, University of Victoria, Victoria BC, Canada; 2 Department of Gender Studies, University of Victoria, Victoria BC, Canada; 3 School of Social and Political Sciences, University of Melbourne and Australia and New Zealand School of Government; 4 Department of Sociology and the Canadian Institute of Substance Use Research, University of Victoria, Victoria BC, Canada

**Keywords:** cesarean section, fear, risk, implementation barriers

## Abstract

In Canada, as elsewhere in the world, caesarean sections are the most common surgical procedure performed in hospitals annually. Recent national statistics indicate 28% of infants in Canada are born by c-section while in the United States that number rises to 33%. This is despite World Health Organization recommendations that at a population level only 10–15% of births warrant this form of medical intervention. This trend has become cause for concern in recent decades due to the short and long-term health risks to pregnant women and infants, as well as the financial burden it places on public health care systems. Others warn this trend may result in a collective loss of cultural knowledge of a normal physiological process and, in the process, establish a new “normal” childbirth. Despite a range of interventions to curb c-section rates—enhanced prenatal care and innovation in pregnancy monitoring, change in hospital level policies, procedures and protocols, as well as public education campaigns—they remain stubbornly resistant to stabilization, let alone, reduction in high-income countries. We explore—through a review of the academic and grey literature—the role of cultural and social narratives around risk, and the responsibilization of the pregnant woman and the medical practitioner in creating this kind of resistance to intervention today.

## 1. Introduction

Globally, there has been a steep rise in caesarean section (CS) rates over the past several decades. While rates vary significantly internationally, some countries report as many as 55% of babies are delivered by CS. At the same time, the World Health Organization (WHO) [1] states CSs should only be undertaken when “medically necessary”—i.e., when necessary to prevent negative health outcomes for infant and/or mother—and that rates higher than 10–15% are not associated with reductions in maternal and newborn mortality rates [1].

In recent years, significant efforts have been made by health care professionals, health and social scientists, policy makers and governments to reduce CS rates; these efforts have included interventions informed by evidence-based research on the social and medical factors that lead to higher CS rates. Strategies to reduce CS rates include educating pregnant women and maternity-care providers about the benefits of spontaneous vaginal birth, as well as approaches to support this mode of delivery, including increasing access to and support for midwife-led maternity care. Other reduction efforts are aimed at the clinical setting, by developing new protocols and policies designed to increase monitoring of decision making processes to reduce CS; these strategies emerge from a growing consensus that care provider discretion is the central driver of higher CS rates [2],[3]. Although these efforts have shown success in specific settings, across many countries CS rates continue to inexorably rise.

The focus of these intervention strategies mirrors the existing literature which tends to distinguish between maternal decision-making processes, often portrayed as fear-based and socially and psychologically determined, and the decision-making of care-providers and policy-makers, which are understood to be informed by technologically mediated and rationally grounded risk-calculi. In contrast to this literature, we argue that *all* decision-making in maternity care is deeply embedded in social and cultural narratives of risk and that this embeddedness represents one of the primary barriers to arresting CS rates. This understanding dissolves the distinctions between indicators for medically necessary CS and *non*-medically necessary or “elective” CSs and provides a more comprehensive perspective of how decision-making processes about mode of delivery are never a straightforward techno-rational process but are instead rooted within socio-historical contexts. Finally, we argue that there is an important disconnect between medical and social understandings of risk, and that addressing CS rates will necessarily require engaging with social narratives of risk, and making sense of how those social narratives play out and are amplified through medical surveillance technologies and responsibilization discourses at the level of policy making, clinical interventions, health provider discretion and maternal choice.

We begin with a brief overview of historical and geographic trends, followed by a review of the grey literature on the successes and failures of interventions to address high CS rates. We then place these interventions within a socio-historical context of risk narratives, surveillance medicine, and responsibilization. We conclude with recommendations for incorporating a more fully social and cultural understanding of risk into maternity care practice.

## 2. Context: The Social Determinants of Caesarean Sections

Debates about the “right” level of CS rates date from the advent of the modern CS in the 1880s [3]. At that time, CS were associated with maternal mortality rates as high as 80% and were mostly reserved as last-hope interventions to save a fetus from a dying or dead woman giving birth [4]. With the introduction of antibiotics, advances in asepsis, as well as blood transfusion, maternal mortality rates dropped to 5–10% by the early 1900s with rates as low as 3–6% by the 1920s in some hospitals [3],[5]. Nonetheless, even while maternal mortality and morbidity rates were improving, the indicators for CS at this time remained quite restrictive, and were generally framed by a concern for the survival of the birthing mother in the context of CS as a high-risk, life-saving intervention [3]. However, pressures to expand indicators for CS emerged very quickly; as Cyr notes, as early as 1906 there were calls to allow for elective CS in cases of “over civilized women in whom the natural powers of withstanding pain and muscular fatigue are abnormally deficient” [3],[6].

At a most basic level, debates about CS rates revolve around finding the right balance between the harms associated with surgical intervention and its benefits. Even today, CS are associated with significant and sometimes permanent short and long-term health risks to pregnant women and infants, especially in settings where obstetric facilities are poorly resourced [1],[7],[8]. Thus, both elective and especially emergency CSs—i.e. where there is an immediate danger to infant and/or the pregnant woman—have poorer maternal outcomes [9]. Greater morbidity includes an increased risk for postpartum hemorrhage, uterine rupture with subsequent births, and urinary tract injury as well as increased pain, infection and longer recovery periods. Women are also twice as likely to be readmitted to hospital after a caesarean delivery for maternal complications [10]–[12].

From a population health perspective, elective CSs “command a disproportionate share of global economic resources”, which in turn has a negative effect on health equality within and between countries [1]. Some regions in Africa, for example, have rates that fall as low as 3% [1]. These rates indicate unavailable or inadequate obstetric care with corresponding high incidence of infant and maternal mortality [13]. Other regions, including Latin America and the Caribbean have rates that far exceed recommendations, including Brazil where 55.6% of infants are born by CS [1],[13] On the surface this suggests that Brazil is relatively well off and has widely accessible obstetric care. A closer look, however, reveals a two-tiered system with relatively high rates of infant and maternal mortality among poor women accessing under-resourced public health care, and a disproportionate number of CSs being performed on those who can afford to purchase private health care [14].

Similar disparities appear in high income countries like Canada and the United States (US) which boast relatively well-resourced health care systems. Recent national statistics indicates the CS rate in Canada is 28%, which is slightly below the North American 32.3% average [15],[16] but 10% higher than it was in the 1990s [17]. In spite of a range of efforts to reduce these rates, and in the face of lower risk profiles in general of the women giving birth, CS rates rise annually in these countries, by an average of 1.6% each year [2],[8] with regional differences in maternal and infant mortality mapping along socio-economic and ethnic-racial lines [8],[18].

The above suggests that CS rates are linked to socio-economic determinants and the broad disparities in wealth that exist within and between countries. At the same time, these economic factors do not explain the variability found within relatively homogenous geographic regions. Research from both the US and United Kingdom (UK), for instance, highlight how rates can vary dramatically between and within regional health authorities. Recent data from California shows that in urban contexts with consistent state level policies aimed at curbing CS rates, and even after controlling for demographic characteristics, hospitals can differ in how many CSs are performed by as much as 32% [19]. Another study from the UK directly observed that midwife-led maternity care teams have CS rates as low as 12–15% while some obstetrician-led private care teams have rates as high as 69% [20]; this study also noted similar differences based on care team even within the same publicly funded health care setting (12–34%) [20]. These results point to the complexity of the drivers increasing CS rates, and the need for analytical frameworks that take into account this complexity. In particular, as we point to below, the CS decision-making context has multiple levels, being simultaneously propelled by a range of factors, including maternal demand, health-care provider discretion, the culture of specific clinical settings and the social context, framed in large part by the decisions of the policy making community who determine the broad health policy directives and frameworks that shape the decisions of all players.

## 3. Interventions to Reduce Caesarean Section Rates

Given the inexorable rise in CS rates in recent decades and widespread concern over the health, financial, and cultural implications of CSs, there have been concerted efforts made since the 1990s to curb increases in the number of procedures. Strategies to reduce the prevalence of CSs that have been evaluated most rigorously in the literature can be divided into those that intervene during the prenatal stage and those focused on the stage of labour and delivery. Prenatal care strategies to reduce rates focus predominantly on the education of pregnant women and families, with some focusing on technological solutions and institutional policies including: antenatal care models, exercise training for pregnant women, education and the management of fear of childbirth among women and their families, change in labour induction policies, structured education for pushing, as well as medical interventions including hyaluronidase injection of the cervix [21]. Strategies to reduce CS that are focused on what happens during labour and delivery focus more on care provider education and training alongside attention to on policy and building awareness of the medicalization of childbirth: promoting natural childbirth, encouraging midwife-led care, active management of labour, psychosocial support, changes in hospital infrastructure to encourage interpersonal engagement over electronic monitoring, as well as other strategies including increased intravenous fluids, pain medications, amnioinfusion, etc [21].

The extensive research on the need to reduce CS has given rise to numerous meta-analyses, most of which have concluded that the available studies lack comparability with objective measurements of performance, as well as, relevant, interpretable data [22]. Further, the majority of this research was conducted among populations of “low risk” pregnant women and aimed at informing the decision-making processes of pregnant women and maternity care providers [21]. However, as these meta-analyses show, most trials done to reduce CS have focused on intervening in clinical guidelines and hospital-level programs and policies [21],[22], reflecting the emerging consensus that health provider discretion and organizational culture are primary drivers of CS rates [2],[19],[22]. As one study reveals, from the perspective of providers, there is no tangible “downside” to high caesarean rates [19]. However, this focus of the literature may also reflect a methodological bias wherein it is easier to measure changes in the decision-making practices of a more discrete population (maternity care providers). At the same time, the decision to focus on clinician discretion is supported by ample research that shows patient education as a stand-alone strategy does not change behaviour over the long term. One study in Australia found, for instance, that educational efforts—including the dissemination of information and empowerment pamphlets and the provision of a peer-support network—had little effect on CS rates overall and may, in fact, be counterproductive as they can increase women's fears surrounding childbirth. These researchers further note that younger, and less educated women were more resistant to education, instead preferring to defer to the expertise of their care provider [23]. Older and more highly educated women showed greater interest in increasing their knowledge but were more selective, and integrated the information they chose with their own belief systems [24]. Similarly, a meta-analysis reviewing 16 studies to determine the effect and safety of non-clinical strategies in reducing non-medically necessary CS, including the education of pregnant women alongside other strategies aimed at clinicians, found that few educational efforts aimed at expectant mothers were shown to decrease CS effectively [23].

As would be expected, the literature indicates that the most effective strategies to reduce CS are multifaceted and targeted toward multi-stakeholder efforts, such as establishing best practice guidelines informed by the expertise of national and international agencies and expert organizations, audits of hospital-level policies and practices with feedback on specific changes and improvements (e.g., hospital and physician benchmarks and public report cards), hospital repayment and malpractice reform, as well as the identification of specific barriers with concrete actions to address those barriers [22]. It has been shown that specific clinical environments can present specific challenges in the implementation of an intervention by fostering local decision-making cultures [22],[25] that favour CS over vaginal deliveries. A Canadian study, for instance, found that clinicians were “more sensitive to maternal and fetal health during trial of labour” and inclined toward CS which they believed were more expedient and posed fewer risks [26]. These clinicians further identified maternal demand as a barrier, citing women's rejection of vaginal birth after caesarean delivery (VBAC) due to their fear of the pain, morbidity, and mortality associated with childbirth, both for themselves and their newborns. At the same time, clinicians noted they were less willing to educate women about the risks and benefits of VBAC as they found it more time-consuming than the education they provided in advance of a CS [22]. Here we see obstetricians understanding their own decision-making processes as a cost/benefit analysis, underwritten by their own understanding of the relative risks—a discourse that elides how they practice a form of defensive medicine out of fear of malpractice and litigation [19],[22].

At the same time, it is clear that clinical environments and the culture of physician decision-making represents only one aspect of this discussion. For instance, a meta-analysis comparing midwife-led models of care with other models of care for child-bearing women and their infants found that while midwife-led care meant fewer obstetric interventions, fewer adverse perinatal outcomes, and in general better health and wellness for mothers and infants, it did not reduce CS rates overall [27].

In sum, while there are examples of successful interventions in specific settings, there has been little achieved in harnessing these interventions to a substantial reduction in global CS rates in the 21^st^ century [21]. While numerous studies have focused on a range of non-clinical factors linked to higher CS rates as well as resistance to change, the role of social and cultural risk narratives as part of the clinical environment has been significantly understudied. Instead, studies of risk, or fear, as a psycho-social concept (and as opposed to a medical or biological profile) tend to focus on pregnant women and not on physicians, midwives or the maternity healthcare policy community [28]. Conversely, fear of litigation is regularly referred to in the literature on physician discretion, but as a rational response to structural and organizational constraints on physician decision-making, as opposed to embedded within social context. Next, we provide an overview of risk as a socio-medical concept, and how risk narratives alongside surveillance medicine has shaped maternity care practices and the decision-making context for women, their families, and care providers.

## 4. Surveillance medicine, risk, and responsibilization

Since the early 20^th^ century, medical knowledge and medical practice in high-income countries has been increasingly organized around two intersecting ontological/epistemological pillars: risk and surveillance [29],[30]. Both are deeply embedded in socio-historical context, and are transparent to a range of other cultural and social concerns, including sexuality, gender, race, as well as socio-economic status [29],[31]. This social nature of medical epistemology has been widely documented, in particular as pertains to maternity care.

The concept of risk has currency far beyond medicine, with even the most intimate human experiences subject to risk analysis [32],[33]. Risk operates simultaneously on multiple registers; at a structural level, risk refers to the uncertainties and contingent outcomes associated with large systemic events—ecological disasters, terrorist attacks, and global recessions [34]. At a psycho-social level, risk also speaks to a world orientation, one in which risk assessment and avoidance are paramount at all times; as Giddens has noted, risk is also an orientation to action that magnifies and amplifies actual danger and risk [32],[35]. In particular, risk is never solely an impartial assessment of harm, but rather, identifying risk always involves assessing and prioritizing some harms over others [34]. Finally, risk, in the Foucauldian tradition, as outlined by Dean is “a way of ordering reality and rendering it calculable” [36].

In medical practice, risk refers to the scientific probability of a specific outcome occurring. Risk in this context is ostensibly determined by gathering population data and making objective calculations of risk based on phenomena that exists outside the bounds of what is considered “normal” [37]. In biomedicine, clinicians and statisticians develop risk calculi on the basis of statistical probabilities drawn from historical data. Their evaluation of risk thus relates to determining probabilities in relation to morbidity and mortality with the aim of carrying out measures to reduce pain, illness, and injury [38]. While such data is often presented as objective knowledge, there is considerable disagreement regarding the validity of these kinds of analyses, in part because of the role personal knowledge, experience, and or judgment plays in interpreting such data is rarely reflexively considered [39]. Further, there is often a fallacy of misplaced scale whereby the risk that an individual has for a certain outcome is assumed to be the same as that occurring at a population level; this is a level of complexity that does not easily translate into medical practice [40].

Risk-based medical practice operates through, and in conjunction with, surveillance medicine. Surveillance medicine refers to the increasing use of diagnostic technology, as well as epidemiological and statistical models to create probabilistic models of health risks [41]; at the same time, surveillance medicine has contributed to an ontological shift whereby categorical understandings of health and illness (i.e., being healthy is mutually exclusive from being ill) to a more ordinal, or scaled understanding, that frames healthy populations from the perspective of their potential for illness [42]. Surveillance medicine thus problematizes normal health as a status of “low-risk” that requires constant monitoring and self-discipline (responsibilization) in order to maintain itself. As Kringeland and Mölller have summarized, life itself is increasingly posited as a threat to health [38].

In the context of maternity care, surveillance and risk medicine have to be understood within a set of important demographic, historical, and cultural factors. In the 19^th^ and early 20^th^ centuries risk was understood primarily in terms of moral and psychological risks, but was also coloured by conceptions of the lack of personhood of the fetus. In other words, risk was understood predominantly in terms of dangers to the pregnant woman; to the extent that these risks were transferred onto the fetus, this occurred because the fetus was transparent to the mother's physical, emotional, and moral health [42]. Today, advancing maternal age, falling birth rates, and the increasing relevance of fetal personhood in legal and cultural understandings of pregnancy (driven as well by improvements in diagnostic and fetal imaging technologies) have contributed to an environment in which the health of the fetus is seen as paramount and independent of the mother's health, and in which competing claims to personhood of the pregnant woman and the fetus are also part of the landscape of risk [42]–[45].

The above illustrates that risk in maternity care, as in other contexts, operates on a number of registers: as part of a “risk profile” of a pregnant woman, but also as an orientation to maternity care and as a cultural and social narrative, in which risk identification, avoidance, and in general “risk talk”, are paramount features of maternity care. As we discuss further below, risk narratives shape women's own understandings of their pregnancy, that of partner or family members, *as well as* the actions and decisions of the care providers that they work with, the policy community who shape maternal health decision making frameworks and how care providers understand their own safety in litigious environments.

## 5. Risk narratives and decision-making in maternity care

### 5.1. Narratives of risk: women's bodies and maternal demand

While there are historically located and socially mediated narratives of risk that underwrite *both* maternal and health provider decision-making processes, these narratives in pregnancy are not objective, free-floating measures of statistical probability of adverse outcomes. Instead, risk narratives are always imbued with other social and cultural expectations about the female body; in other words, the biomedical framing of a risky pregnancy is embedded in a particular social context [46],[47]. For instance, while it is almost axiomatic today to identify age as a risk factor for adverse outcome in pregnancy, this is a relatively recent phenomenon, and illustrates, among other things, that concerns about the aging mother cannot be understood outside of normative expectations regarding reproductive timing on our understandings of pregnancy and motherhood [42]. Secondly, risk calculi and narratives are not the sole prerogative of physicians and other maternity-care providers but are also deployed by women themselves, partner and family members, and the maternal health policy community. Furthermore, and as discussed further below, risk is a socially authoritative language that is embedded within power dynamics and relationships between health professionals and women [47],[48]. However, the authoritative nature of the language of risk obscures how decision-making is based on social rather than scientific knowledge [40]. Risk narratives are thus multilevel, power-laden, and multidirectional, and in this saturate the decision-making context in maternity care.

If we consider labour, for example, the average length of time it “should” take is six to eight hours. After this period, medical practitioners get concerned about the risks to both the expectant mother and infant and a diagnosis of “failure to progress” may be made [39],[49],[50]. This sets off a series of interventions to medically manage and ameliorate those risks. This kind of techno-rationale thinking discursively saturates our thinking and informs many of our day-to-day decisions and yet, is fraught with errors. Using the example above, historians have noted how the acceptable time for a labour to progress has grown progressively shorter over recent decades without any clear medical indication for why, and that the culprit may be a general “impatience with the labor progress”, particularly in contexts where there are competing demands on physician's time [19]. They further suggest the discursive use of words like “failure”, with the inference that women are “poorly designed”, has fundamentally changed how pregnant women and health professional think about and practice childbirth [49],[50]. The result has been a loss of cultural knowledge of birth as “a positive, life-affirming rite of passage to a dehumanized, mechanistic process” [51].

The importance and role of socially mediated understandings of risk and risk calculi become particularly apparent in the scholarship focusing on elective CSs, especially those described in terms like “maternal demand.” Reducing maternal demand for CS has been a significant focus of the medical scholarship on increasing CS rates. This is despite the definitional problems in the literature on what constitutes maternal demand for CS, particularly in contexts where physicians are responsible for the actual decisions. For instance, the available research in Canada, the UK, Sweden and Australia indicates that only 6–15% of women indicate a preference for CS, but that this preference is heavily informed by the idea that a CS means better, higher quality maternal care and is therefore the most responsible decision. Further research questions the actual numbers of women ostensibly choosing CS, as few hospital records document maternal preference for delivery, or speak to the decision-making process leading up to the delivery. Instead, the data that is collected only speaks to the number of scheduled elective CSs that are performed in hospitals. This data indicates that many of these CSs are performed at the instigation of the physician for reasons unrelated to medical indication, and do not therefore fall under the category of maternal request [28],[52].

What we do know about maternal demand as a driver of higher CS rates highlights the role of risk narratives in shaping women's experiences during pregnancy and the birth decision-making context for women. This context includes for some a general perception that vaginal delivery is risky. These perceived risks include urinary incontinence, vaginal prolapse and/or sexual dysfunction, despite the fact that these are not clearly linked outcomes [10],[53]. Others have noted that women fear the pain associated with labour, and see it as inherently dangerous. A recent exploratory study done in Australia with a sample of 210 women, for example, found that women who were having normal healthy pregnancies spoke about vaginal birth with:

[…] a sense of ambivalence, if not distaste, for the value of vaginal birth as a natural, important and significant life process. This is combined with what appears to be a distrust of the body's ability to undertake labour and safely birth a baby. Constructing the pregnant body as a vessel and birth as ‘getting’ a baby, that holds no intrinsic value and necessitates no active participation, reflects a disconnection between the self and the body, and places control outside the self [14:389].

These women saw CS as offering a sanitized and controlled birth and a “guaranteed” healthy baby, in which they were able to shift the onus of safely delivering their baby away from their bodies and onto their health care professional [14]. Despite CS carrying greater absolute risks, it is increasingly seen as safe by women, in part because it is seen as more predictable [14]. In this case, risk calculi privilege the minimization of uncertainty over safety.

However, the role of risk calculi, and risk narratives in shaping maternal demand for CS in high-income nations should also be understood within demographic and socio-economic contexts and the increasing medicalization of health events [54],[55]. Demographic factors include higher age at first pregnancy, more reliance on assisted reproductive technology and smaller families. Older first-time mothers tend to have higher levels of education than younger mothers [56]; many older mothers have experienced difficulty in conceiving naturally, have experienced miscarriages and some may be giving birth with the expectation that they will not have another child. For women in these categories pregnancy and childbirth is simultaneously an empowering yet often highly anxiety-ridden phenomenon [57]. In a context in which pregnancy and birth are risk-laden, the delivery process is reframed as unimportant; at the same time, this framing casts doubt on the body's natural ability to undertake labour [14].

This is a rationale that becomes especially salient when we consider the ways in which women alone are seen as shouldering the responsibility of birthing a healthy infant. Mitchell has identified the way in which women shoulder this responsibility, and subsequently assume much of the blame when pregnancies go wrong. Mitchell reflects that, “When I asked women in Montreal about risk during pregnancy, the list of potential harms emanating from a woman's body is long. When I asked about risk posed by the father… many regarded the question as puzzling or nonsensical” [58].

Even when women are well aware of the clinical risks associated with CS, they may still choose this option as a way to ameliorate fear and responsibility, or to position themselves as “good” mothers making responsible choices. As noted by Craven, pregnant women who challenge dominant biomedical knowledge around childbirth and mothering are often castigated, and accused of placing themselves and their babies at undue risk [59]. These stigmas are underwritten by the authority that we give to professional discourses in general, but perhaps most especially by authoritative discourses around risk and the ways those link to responsibilizing discourses [60]. In addition, when care providers engage in “risk talk” (which includes categorizing women as high or low risk, identifying for them the statistical likelihood of specific complications occurring and the concomitant risk associated with each intervention), they also engage in more broad generalizations about the relative risks of every behavior [61]. Such talk creates a climate of fear and anxiety in which the “moral weight” of pregnancy creates an overwhelming sense of responsibility where each decision is fraught with consequence [17]. This can initiate a self-perpetuating cycle whereby women are counseled to closely monitor their bodies and behaviours during pregnancy, and submit themselves to increasing medical management, which in turn often translates into a series of interventions, including CS [40]. Feminists have argued that within this environment there is a “supervaluation” of science and technology which leads, unintentionally, to the idea that natural childbirth is an unpredictable process and that the female body is inherently faulty [49]. This discourse elides the absolute risks associated with surgical intervention as compared to spontaneous vaginal birth.

Finally, central to this discussion of what drives the phenomena of maternal demand for CS is debate over the level of autonomy women have in making informed decisions about mode of delivery. This autonomy is exercised in a context shaped by authoritative medical technologies and apparatus [62] and, in many cases, through discursive assemblages of risk that operate at micro and macro levels of medical management from the clinical encounter to hospital and state-wide policy [61]. Within this setting, both women and maternity care providers are seen as having limited agency in exercising choices that align with their beliefs and values. For pregnant women, if they exercise their agency and choose an unassisted or home-based birth, for example, there are real concerns they will be ostracized and labelled ‘bad’ decision makers, and ultimately, bad mothers unwilling to sacrifice for the health of their baby [63]. Few want these fears tested in the event that maternal and/or infant complications do occur. For maternity care providers, especially those ideologically committed to natural childbirth, the heightened sense of accountability that emerges from increasingly strict institutional policies and protocols increases their own sense of fear and anxiety and can undermine their commitment to women's ability to give birth spontaneously [48].

### 5.2. Care provider discretion: decision rules, risk profiles and the social basis of risk

As we discuss above, risk narratives create a socially authoritative language, embedded within and reproduced through power dynamics and relationships between medical professionals and women [47],[48]. This authoritative language of risk obscures the extent to which birth decisions, whether shaped by maternal demand or clinician decision, are made on the basis of social as opposed to scientific knowledge [40]. Below we discuss how risk, as a social narrative, is also at play within the decision-making context of care providers, as well as the maternal health policy community.

One aspect that bears mentioning is what Dahlen and Homer refer to as “litigation-based practice” [64]. Here, physicians encourage or initiate CS not necessarily because the reimbursement is higher, or that they are less time intensive, but also, importantly, because of the fear of medical malpractice lawsuits. Litigation-based practice specifically refers to practices through which “birth risks are managed through adherence to (and sponsoring women's compliance with) ‘active management’ protocols and procedures, to reduce professional and organizational exposure to medico-legal risk” [51].

Such concerns are not limited to obstetricians but increasingly extend to other maternity care providers, including midwives. Scamell writes about how in previous decades it was the job of midwives to manage the “first order” physical health and safety risks associated with pregnancy and birth, but that increasingly their “object focus” of risk has changed and expanded to include their own reputation and that of the organizations they align themselves [48]. The result is that an important aspect of midwifery—i.e., to normalize birth as a natural physiological process—is often superseded by organizational demands, and in particular, “risk technologies”.

In assessing the role of risk narratives in shaping the decisions of maternity care providers it is important to note it is not so straightforward to distinguish between emergency (or medically necessary) CS and non-medical indicators. Cyr [3] argues that all CS decisions ultimately come down to an individual decision point. Furthermore, these decisions are always heavily value-laden and context specific and involve assessing and weighing the probability of risks to both the pregnant woman and the fetus, but also a range of other factors. A helpful concept here is that of “decision rules” and how decision rules are shaped by perceptions of risk [65]. Decision rules identify triggers for action, and limits, for instance, for the length of time a woman is allowed to labour before intervening. However, decision rules vary by risk profile and risk assessment.

Risk profiles of birthing mothers emerge from social understandings. In turn, socially derived risk profiles shape the assessment of risks to the pregnant woman, the infant, as well as risk of litigation. For instance, in the context of mental health, social work, and criminology, scholars have theorized risk assessments as a form of governance: risk profiles and assessments make predictions about the probable future behaviour of an individual on the basis of a statistical profile of the population that an individual belongs. In this, risk profiles set certain individuals up for extra scrutiny and surveillance [33],[41],[62],[66]. It is important thus to ask the question of how social understandings and narratives of risk shape not only risk assessment in prenatal care, but also, importantly, how these risk assessments set into motion surveillance, scrutiny, and attention that shape health providers' decision rules and lead to a higher probability for surgical intervention [17],[65]. There is, for instance, a sizable literature showing that social stigmas, associated with a range of characteristics such as obesity, socio-economic status, disability, substance use (to name just a few) can impact the quality of care that an individual receives [24],[67],[68]. Stengel [69] has linked stigmas associated with substance use during pregnancy to poorer outcomes, driven in part by a reluctance of these women to seek timely maternity care [70].

However, the question of how these social understandings of risk shape physician decisions to conduct a CS has not been comprehensively pursued, especially outside of the sociological literature on stigma, risk, and health. A key-word search of four mainstream scholarly publications specializing in obstetrics (International Journal of Obstetrics and Gynecology, American Journal of Obstetrics and Gynecology, Human Reproduction Update, and Journal of Obstetrics and Gynecology in Canada) found no articles examining the social basis of risk assessment as part of the decision-making context leading up to a CS. This speaks to a significant disconnect between the sociological and medical literatures on the factors driving increases in CS rates in high-income countries.

## 6. Discussion and Conclusion: Social narratives of risk and CS rates

Over the last century, pregnancy and childbirth have become significantly safer with far fewer net negative outcomes; however, people's sense of fear and anxiety about childbirth has only continued to increase [14]. Beck refers to this as the paradox of living in a “risk society” [33] whereby in our attempts to identify and control risks through technological innovation and surveillance, our anxiety inadvertently gets heightened, and in the process, we create more risks for ourselves. This is arguably nowhere more evident than in maternity care, and in particular, in understanding the paradoxical rise in CS rates in high-income countries over the late 20^th^ and early 21^st^ century.

There is an emerging scholarly consensus that the increase in CS rates in high-income countries is largely driven by non-clinical factors—i.e., that the higher incidence of CS does not reflect clinical changes in the population of women giving birth [2]. For the most part, this literature focuses on two drivers of increases in rates of elective CS: maternal demand and health provider discretion, although there is a smaller literature looking at the role of organizational and institutional characteristics in determining variations in CS rates across different hospitals and/or care provider teams. The role of social narratives of risk is variously emphasized across these literatures; in terms of maternal demand, both fear and perceived risk are understood to inform and shape women's perceptions of the birth process, and in particular, their responsibilities to mitigate that risk. This literature often explicitly acknowledges that the risk perceptions of women (as well as fear) are social phenomena that are embedded in particular socio-historical contexts. Conversely, the literature on health provider discretion and decision-making, while acknowledging that these decisions are often based on non-clinical factors, has not explicitly examined social understandings of risk.

We have argued above that both maternal demand as well as physician and other care-provider decision-making should be understood within the context of social narratives of risk that operate at the structural level. Social narratives of risk share several important features that are significant to the birthing context and the decision to have a CS. First, social narratives refer to risk as a quantifiable and statistical property associated with behaviours, physical attributes, and genetic propensities. It is important to note here that while risk statistics almost always refer to populations, as a social narrative, risk responsibilizes the individual: for example, the knowledge that there is a higher incidence of complications during birth for older first-time mothers, is translated to mean that later-in-life motherhood is a “risky” and therefore morally questionable choice [42].

Second, and concomitant with this, risk as a social narrative emphasizes that it is predictable, and thus avoidable; it also places trust in authoritative medical discourses (although narratives that reject mainstream medicine also refer to language of risk). Risk narratives thus counsel birthing women as well as physicians and other care providers to place all their decisions and actions within a responsibilizing risk-avoidance frame [48],[71]. As Scamell writes, “childbirth risks should never be conceived as being self-evident or as impartial, scientific calculation of potential hazard” given that no matter how tenuous the probability, health practitioners after being made sensitive to a particular risk “are professionally bound to persuade the women in their care that these risks not only warrant concern but also demand technological surveillance and management” [71]. Third, social narratives of risk are always embedded in, and reflect, normative assumptions around gender, race, class, among others. An educated choice for one woman can become a risky choice for another, depending on the background assumptions that underwrite our understandings of risk.

A perspective on risk as a social narrative dissolves the distinction between “medically necessary” and “elective” and suggests all decision-making processes about mode of delivery are embedded within this risk-saturated context [39],[63]. However, whereas the social aspect of risk has been fruitfully explored in the sociological and critical health literatures, there has been little engagement with this concept at the level of clinical interventions, health provider discretion and policy making analysis. To do so here would be beyond the scope of this paper; however, we end with a recommendation for further research on how a social understanding of risk can be integrated into clinician practice as well as interventions intended to reduce CS rates. In particular, there is a need to unpack more clearly how social and cultural assumptions inform and underwrite clinical risk profiles, and second, examine how these risk assessments and profiles create conditions of scrutiny and surveillance that may trigger different decision rules and choices by both pregnant women, practitioners and policy makers.
